# Routine human papillomavirus genotyping by DNA sequencing in community hospital laboratories

**DOI:** 10.1186/1750-9378-2-11

**Published:** 2007-06-05

**Authors:** Sin Hang Lee, Veronica S Vigliotti, Jessica S Vigliotti, Suri Pappu

**Affiliations:** 1 Department of Pathology, Milford Hospital, Milford, Connecticut, USA

## Abstract

**Background:**

Human papillomavirus (HPV) genotyping is important for following up patients with persistent HPV infection and for evaluation of prevention strategy for the individual patients to be immunized with type-specific HPV vaccines. The aim of this study was to optimize a robust "low-temperature" (LoTemp™) PCR system to streamline the research protocols for HPV DNA nested PCR-amplification followed by genotyping with direct DNA sequencing. The protocol optimization facilitates transferring this molecular technology into clinical laboratory practice. In particular, lowering the temperature by 10°C at each step of thermocycling during *in vitro *DNA amplification yields more homogeneous PCR products. With this protocol, template purification before enzymatic cycle primer extensions is no longer necessary.

**Results:**

The HPV genomic DNA extracted from liquid-based alcohol-preserved cervicovaginal cells was first amplified by the consensus MY09/MY11 primer pair followed by nested PCR with GP5+/GP6+ primers. The 150 bp nested PCR products were subjected to direct DNA sequencing. The hypervariable 34–50 bp DNA sequence downstream of the GP5+ primer site was compared to the known HPV DNA sequences stored in the GenBank using on-line BLAST for genotyping. The LoTemp™ ready-to-use PCR polymerase reagents proved to be stable at room temperature for at least 6 weeks. Nested PCR detected 107 isolates of HPV in 513 cervicovaginal clinical samples, all validated by DNA sequencing. HPV-16 was the most prevalent genotype constituting 29 of 107 positive cases (27.2%), followed by HPV-56 (8.5%). For comparison, Digene HC2 test detected 62.6% of the 107 HPV isolates and returned 11 (37.9%) of the 29 HPV-16 positive cases as "positive for high-risk HPV".

**Conclusion:**

The LoTemp™ ready-to-use PCR polymerase system which allows thermocycling at 85°C for denaturing, 40°C for annealing and 65°C for primer extension can be adapted for target HPV DNA amplification by nested PCR and for preparation of clinical materials for genotyping by direct DNA sequencing. HPV genotyping is performed by on-line BLAST algorithm of a hypervariable L1 region. The DNA sequence is included in each report to the physician for comparison in following up patients with persistent HPV infection, a recognized tumor promoter in cancer induction.

## Background

Human papillomavirus (HPV) testing was introduced to compensate for the poor sensitivity and specificity of the Pap smear cytology often used as diagnostic tool for borderline precancerous lesions [1]. Digene Hybrid Capture 2 (HC2) test, the only test approved by the U.S. Food and Drug Administration (FDA), is commonly used to determine if a cervicovaginal cell suspension contains "high-risk" oncogenic HPVs [2], often functioning as a triage for colposcopic evaluation of the cytologically borderline cases [3-5]. However, it is now recognized that persistent infection of a "high-risk" HPV, not the mere presence of the HPV virus itself, is the pivotal promoter in causing cervical precancerous lesions and cancer [6-9]. Most of HPV infections, even caused by "high-risk" genotypes, are transient with normal Pap cytology in sexually active young women [10-13]. In 93% of initially infected women, the same viral type is not detected upon re-examination four menstrual cycles later [14]. The median duration of positivity detectable by PCR for a specific HPV type in these young women is 168 days [15]. Multiple "high-risk" HPV infections do not constitute a higher risk for the development of cervical neoplasia when compared with single persistent high-risk HPV infection [16]. For the development and maintenance of a high-grade squamous intraepithelial lesion (SIL), the risk is greatest in women positive for the same genotype of HPV on repeated testing [6-8]. Viral load is not a useful parameter to predict high-grade SIL [17]. High-grade SIL is often associated with a viral DNA load lower than that observed in less severely affected cells [18].

In view of the recent advance in the understanding of the relationship between persistent HPV infection and cervical neoplasia, a sensitive and specific technology to detect and accurately genotype HPV is needed for clinical management of persistent infections. The HC2 test cannot be converted to a genotyping assay and is associated with a significant number of false-negative and false-positive results when compared with other more stringent PCR-based HPV genotyping assays [19-23]. It is reported to generate 25% false-negative results in cases with biopsy-proven high-grade SIL even when all these biopsies have been proven to contain high-risk HPV DNA by PCR [24]. "The lack of multiple, competitive, well-validated tests" for HPV assay is quoted as being "a problem" in formulating new guidelines in management of cervical abnormalities [25].

The introduction of the type-specific Gardasil™ HPV vaccines into the sexually active female population also requires genotype monitoring of the HPV infections before and after immunization to develop prevention strategy for the individual patients. Based on a "Background Document" submitted to the FDA [26], injection of HPV vaccines into women who have concurrent vaccine-relevant HPV type infections may increase the risk, by 44.6%, of developing high-grade precancerous lesions in the cervix. Therefore, it would be prudent to perform a genotype-specific HPV assay if prior HPV infection is suspected.

Target nested PCR amplification of a conserved region of the HPV L1 gene DNA with the consensus MY09/MY11 and GP5+/GP6+ primers, or their equivalent, followed by genotyping with direct DNA sequencing is a generally accepted scientific tool in research [21,22,27]. However, it has not been used in clinical laboratories because handling the temperature-sensitive PCR reagents and the requirement for template purification in the PCR protocols are too labor-intensive for routine applications. This paper records our experience in using a high-processivity, room temperature-stable robust DNA polymerase system to facilitate the transfer of this molecular technology into clinical laboratory practice. Each HPV-positive result is validated by genotyping with direct DNA sequencing of a hypervariable region of the L1 gene. The HPV DNA sequencing information can be included in the laboratory report for future clinical follow-up of persistent infections.

## Methods

Since the PCR conditions adapted for this protocol depended on the use of a new low-temperature ready-to-use moderately thermostable DNA polymerase system, the sensitivity and specificity of the LoTemp™ HiFi^® ^DNA polymerase in amplification of HPV DNA were first validated and compared with those obtained by standard heat-resistant *Taq *polymerases available on the market.

The purified full-length plasmid DNAs of HPV types -16, -18 and -6B purchased from American Type Culture Collection (ATCC) were used as standards for method development. Then the HPV type-16 DNA was used as a routine positive control. Molecular grade pure water instead of DNA extract was used as negative control.

Theoretical sensitivity of the PCR system chosen for this study was determined by using serial 10-fold dilutions of the ATCC-certified HPV standard containing 200 ng of plasmid DNA of HPV-16, -18 and -6B with TE buffer to single copy of genomic DNA per μL as the template to run primary and nested PCR on each dilution in duplicate. The theoretical number of copies of HPV DNA in the template used for each MY09/MY11 PCR was calculated according to a generally accepted conversion formula [28]. All nested PCR products were confirmed by DNA sequencing to be those of respective HPV genotypes expected.

For *Taq *PCR amplifications, the reaction mixture of 25 μL contained 100 mM KCl, 20 mM Tris-HCl pH 8.0, 25 mM MgCl_2_, 2.5 mM of each dNTP, 2.5 units of Takara Taq polymerase (Takara Bio Inc., Shiga, Japan), 100 pmol of each consensus primer (MY09/MY11 or GP5+/GP6+) and 1 μL of HPV DNA at various dilutions in TE buffer (or if for nested PCR, 1 μL of the MY09/MY11 PCR products). The reaction mixture was subjected to 35 cycles of amplification in an MJ Research thermocycler (Waltham, MA). Each cycle consisted of a denaturing step at 94°C for 0.5 min, an annealing step at 55°C for 1 min, and a chain elongation step at 72°C for 1 min.

In LoTemp™ HiFi^® ^DNA polymerase PCR, the protocol described below for clinical specimens was followed except that a 35-cycle amplification was used for comparison with *Taq *PCR amplification.

The clinical samples used for this study were 515 alcohol-preserved liquid-based cervicovaginal cytology specimens (Cytyc or Surepath) submitted by physicians in the New Haven, Connecticut area as part of routine gynecologic examinations. Age distribution of the patients and the cervical pathologic conditions were not the subjects of this study. Digene HC2 test for high-risk HPV ordered by the physicians was performed routinely on each sample by one of the two independent clinical laboratories (Quest Diagnostics Laboratory, Wallingford or Pathology & Laboratory Services, LLC, Woodbridge, CT) according to instructions provided by Digene Corporation (Gaithersburg MD). In general the patients were women below age 30 who had a Pap cytology diagnosis of atypical squamous cells of undermined significance (ASCUS) or women 30 years and older regardless of the Pap cytology findings [4].

After the material was taken from each sample for routine cytology and HC2 test, about 1 mL of the cell suspensions was placed in a 1.5 mL Eppendorf tube, blind-coded with a case number and transferred to the laboratory at Milford Hospital for HPV PCR/DNA sequencing.

DNA extraction from the alcohol-fixed cells was accomplished according to a National Cancer Institute (NCI) protocol [29] with minor modification. Briefly, the cell suspension was first centrifuged in an Eppendorf microcentrifuge (model 5424) equipped with a rotor (model FA45-24-11) for 5 min at 13,000 rpm. The cells in the pellet were washed in 1 mL reagent grade water and then in 1 mL buffer consisting of 50 mM Tris-HCl, 1 mM EDTA, 0.5% Tween 20, pH 8.1. The washed cell pellet was re-suspended and digested at 45–55°C overnight in 100 μL of 0.1 mg/mL proteinase K (Sigma Chemical Co., St. Louis, MO) dissolved in the same washing buffer. After denaturing the proteins in the cell digestate in a metal block heated to 95°C for 10 min and a final centrifugation of the digestate at 13,000 rpm for 5 min, the supernatant was carefully pipetted out and placed in a clean microcentrifuge tube to be used for PCR without further purification or stored at -20°C.

The general methodology of primary PCR amplification of a 450 bp segment of the HPV L1 gene with a pair of consensus MY09/MY11 primers followed by nested PCR with a pair of GP5+/GP6+ general primers was used for HPV DNA preparation. The 150 bp nested PCR products in the positive specimens were genotyped by direct DNA sequencing [21,22] with minor modifications briefly summarized as follows.

For primary PCR amplification, 1 μL of the DNA extract, 1 μL of 10 μmolar MY09 primer, 1 μL of 10 μmolar MY11 primer and 2 μL of water were added to a PCR tube containing 20 μL of LoTemp™ HiFi^® ^DNA polymerase ready-to-use mix (HiFi DNA Tech, LLC, Trumbull, CT) which contains all the components needed for low temperature PCR, including dNTPs, Mg^++^, buffer, HiFi^® ^DNA polymerases, proprietary dsDNA melting agents and dNTP preservatives, to reach a final 25 μL reaction volume. For thermocycling, the temperature steps of a TC-412 Thermal Cycler (Techne Incorporated, Burlington, NJ) were programmed for an initial heating at 85°C for 2 min, followed by 30 cycles at 85°C for 30 sec, 40°C for 30 sec, and 65°C for 1 min. The final extension was 65°C for 10 min.

For nested PCR, a "trace" of the MY09/MY11 PCR products was transferred by a glass rod with clean wettable surface of about 1.5 mm in diameter to a second PCR tube containing 25 μL of complete nested PCR reaction mixture consisting of 20 μL of LoTemp™ HiFi^® ^DNA polymerase ready-to-use mix, 1 μL of 10 μmolar GP5+ primer, 1 μL of 10 μmolar GP6+ primer and 3 μL of water, using the same thermocycling program as described above.

After completion of the primary and the nested PCR, a 5 μL aliquot of the PCR products was pipetted out from each tube and mixed with 2 μL loading fluid for electrophoresis in a 2% agarose gel containing ethidium bromide. The gel was examined under UV light. Visualization of a 450 bp PCR product band in the MY09/MY11 lane and/or a 150 bp band in the nested PCR lane on the agarose gel provided evidence of HPV DNA in the sample, pending genotyping with direct DNA sequencing as a means of final validation.

For DNA sequencing, 1 μL of the nested PCR products, if positive, was pipetted out from the nested PCR tube for direct DNA sequencing, using 1 μL of 5 μmolar GP6+ primer as the sequencing primer, 1 μL of the BigDye^® ^Terminator (v 1.1/Sequencing Standard Kit), 3.5 μL 5× buffer, and 13.5 μL water in a total volume of 20 μL for 20 enzymatic primer extension/termination reaction cycles in an ABI thermocylcer Model 9600 according to the protocol supplied by the manufacturer (Applied Biosystems). After dye-terminator cleanup with a Centri-Sep column (Princeton Separations, Adelphia, NJ), the reaction mixture was loaded in an automated ABI 3130 four-capillary Genetic Analyzer for sequence analysis. Sequence alignments were performed against various standard HPV genotype sequences stored in the GenBank database by on-line BLAST analysis to arrive at specific genotyping.

One μL of each DNA extract was placed in a separate PCR tube with a β-globin primer pair [30] for human genomic DNA amplification as a control of specimen adequacy. The primers for β-globin gene amplification were 1 μL of 80 μmolar 5'-ACACAACTGTGTTCACTAGC and 1 μL of 80 μmolar 5'-CAACTTCATCCACGTTCACC in a 20 μL of LoTemp™ HiFi^® ^DNA polymerase ready-to-use mix with 2 μL water added. The LoTemp™ thermoclycling program mentioned above was used.

Three (3) PCR tubes per sample were used routinely for the β-globin gene, the MY09/MY11 primer and the GP5+/GP6+ nested amplification, respectively.

To test performance reproducibility of this PCR/DNA sequencing procedure, aliquots of the digestate of 30 individual clinical samples were tested in parallel duplicate runs. The duplicate results of the three PCRs on each case and the genotyping results of the positive cases by DNA sequencing were compared.

Samples that did not show an MY09/MY11 or a GP5+/GP6+ PCR band, but showed evidence of positive β-globin gene amplification were interpreted as HPV-negative. Specimens that did not show any PCR products in all three lanes were considered unsatisfactory for evaluation due to low DNA extraction or presence of a PCR inhibitor. There were two (2) unsatisfactory cases among a total of 515 processed. The remaining 513 cases were accepted as satisfactory for analysis. Of these 513 cases, 107 were positive for nested PCR products, all proven to be those of HPV DNA by direct DNA sequencing, using GP6+ as the sequencing primer.

The samples infected with more than one genotype of HPV were indicated by the appearance of numerous ambiguous or overlapping peaks in the DNA sequencing tracings. For each of these mixed infections, the nested PCR products were subjected to additional four individual primer extension/termination reactions to rule out infection by the Gardasil™ vaccine-relevant HPV, namely HPV types -16, -18, -6 or -11, using the following type-specific primers [31].

HPV-16 type-specific sequencing primer 5'-GCTGCCATATCTACTTCAGA-3'

HPV-18 type-specific sequencing primer 5'-GCTTCTACACAGTCTCCTGT-3'

HPV -6 type-specific sequencing primer 5'-GTGCATCCGTAACTACATCTT-3'

HPV -11 type-specific sequencing primer 5'-GTGCATCTGTGTCTAAATCTG-3'

All oligonucleotides used as primers for this study were synthesized and purified with the oligonucleotide purification cartridge (OPC) method by the Pathology Department DNA Synthesis Lab, Yale University (New Haven, CT).

To avoid cross contamination, three separate rooms with no air re-circulation were dedicated to nucleic acid amplification tests. Two of the rooms were each equipped with a 32" PCR workstation (AirClean Systems, Raleigh, NC). All pre-amplification procedures were performed in PCR station I. All post-PCR procedures were carried out in PCR station II, including preparations for the nested PCR and sequencing reaction. Gel electrophoresis and DNA sequencing were performed in the third isolation room. No post-PCR materials or any items contaminated by amplicons, or equipment used in the post-PCR rooms were allowed to enter the pre-PCR working space.

## Results

**Figure 1:**
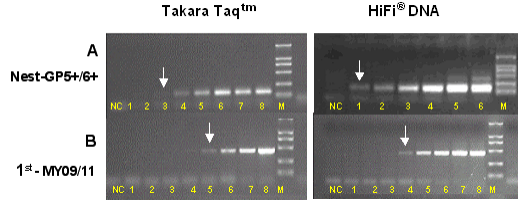
**Comparative amplification of HPV-16 DNA with Taq DNA polymerase and with LoTemp™ HiFi**^®^**DNA polymerase**. Lanes 1–8: HPV-16 plasmid DNA template at 85 × 10^1^, 85 × 10^2^, 85 × 10^3^, 85 × 10^4^, 85 × 10^5^, 85 × 10^6^, 85 × 10^7 ^and 85 × 10^8 ^copies per milliliter. NC: negative control. M: molecular marker. Arrows indicate the lower limit of detection by nested PCR (A) and by the 1^st ^primary PCR (B) with Takara Taq DNA polymerase and LoTemp™ HiFi^® ^DNA polymerase, respectively.

The LoTemp™ HiFi^® ^DNA polymerase was about 10 times more efficient than *Taq *DNA polymerases in amplifying HPV plasmid DNA by MY09/MY11 PCR and about 100 to 1000 times more efficient when the first amplification was followed by a GP5+/GP6+ nested PCR in tandem. The nested PCR technology described in this paper proved to be a sensitive method for the detection of 1–10 copies of purified genomic DNA of HPV types -16, -18 or -6B. But 10^4^-10^5 ^copies of genomic DNA were needed as PCR templates for UV visualization of a positive MY09/MY11 primer amplicon band after electrophoresis (Fig. 1). The specificity of all nested PCR products was validated by HPV genotyping with direct DNA sequencing.

**Figure 2:**
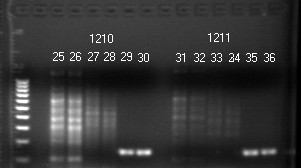
**HPV Nested PCR in Clinical Samples and Reproducibility**. Agarose gel showing PCR products of targeted DNAs extracted from two clinical samples in duplicate. The targeted β-globin DNA amplicon is 110 bp long, as seen clearly in lanes 25 and 26 (#1210), but is hardly visible in lanes 31 and 32 (#1211). Co-amplification of other human genomic DNA fragments and a positive nested PCR amplicon assure specimen adequacy in both samples. Molecular ruler = 100–1000 bp (far left). Lanes 25/26, 27/28, 29/30 = β-globin gene, MY09/MY11, GP5+/GP6+ PCR, respectively-sample #1210. Lanes 31/32, 33/34, 35/36 = β-globin gene, MY09/MY11, GP5+/GP6+ PCR, respectively-sample #1211

Reproducibility of this nested PCR assay in the detection of HPV DNA in clinical specimens was confirmed by running two parallel sets of PCR with a split single sample digestate as the paired templates, including the β-globin gene, the MY primer and the GP nested primer amplifications on each set for 30 HPV-positive cases. Pairs of identical results on electrophoresis gel were obtained in all three amplifications for the 30 split samples (Fig. 2). The nested PCR products obtained on the duplicate sets were confirmed by DNA sequencing to be of the same HPV genotype.

For clinical samples, the primary MY09/MY11 PCR generated a distinct 450 bp product band after 30 repeated amplification cycles in only 37 of the 107 HPV-positive cases detected by nested PCR. More than 65% of the clinical HPV-positive amplicons relied on a nested PCR for UV visualization. Without a nested PCR, the amplicon of the MY09/MY11 PCR was often masked due to co-amplification of other DNA molecules in the clinical samples (Fig. 2).

**Figure 3:**
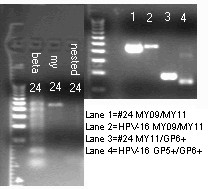
**HPV-39 Heminested PCR (clinical specimen #24)**. Left gel: beta: β-globin gene amplification-specimen #24. my: Positive 450 bp MY09/MY11 PCR product- #24. nested: Negative GP5+/GP6+ nested PCR product- #24. Molecular marker on far left. Right gel: Lane 1: #24 MY09/MY11 PCR product, 450 bp. Lane 2 HPV-16 control Y09/MY11 PCR product, 450 bp. Lane 3: #24 MY11/GP6+ heminested PCR product ~ 195 bp. Lane 4: HPV-16 control GP5+/GP6+ nested PCR ~ 150 bp. Molecular marker on far left

For some isolates, notably those of HPV-39 and HPV-73, the standard GP5+/GP6+ nested PCR failed to amplify the target DNA fragment even when the MY09/MY11 primer PCR amplification was successful. These cases were recognized on the gel electrophoresis, showing a positive 450 bp MY09/MY11 PCR amplicon in the absence of an expected concomitant 150 bp nested PCR product. For these HPV strains, a heminested PCR with MY11/GP6+ primers generated a homogeneous amplicon (Fig. 3) for direct DNA sequencing.

**Figure 4:**
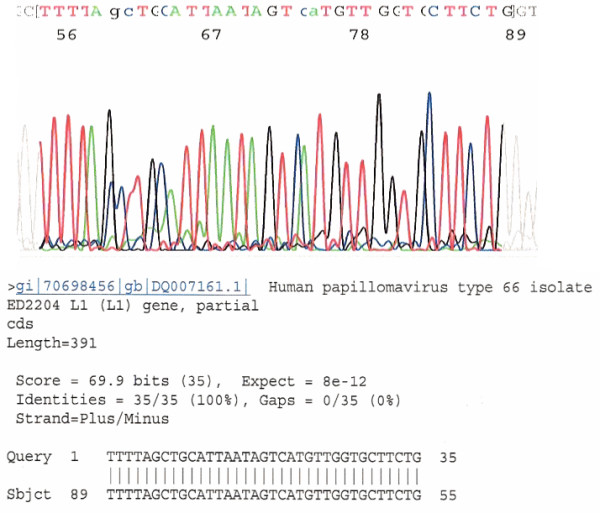
**DNA Sequence of Nested PCR Product Downstream of GP5+ for Genotyping**. This is a typical DNA sequence excised from the color tracing downstream of the GP5+ binding site of the HPV DNA L1 gene. BLAST alignment analysis of a 34 (up to 50) bp sequence of this hypervariable region provides unequivocal evidence for HPV genotype 66 based on the database stored in the GenBank.

After cycle sequencing, BLAST algorithms by alignment of a 34 bp DNA sequence in the hypervariable region of the L1 gene downstream of the GP5+ binding site against known HPV genotype sequences stored in the GenBank database usually determined the genotype of the HPV isolates detected [32]. A 100% "identities" match between the "query" sequence and the "subject" sequence was reached for each final genotyping (Fig. 4). This sequence tracing with its on-line BLAST algorithm for genotyping was incorporated in the report for clinical follow-up of persistent infections. However, HPV-16 has numerous sequence variants, some of which share an identical sequence in this region with some strains of HPV-31 and HPV-33, and required BLAST algorithm of a 50 bp sequence for genotyping distinction.

Of the 513 liquid-based cervicovaginal samples, the nested PCR method detected at least one HPV strain in 107, with an overall positive rate of 20.9%, including 74 cases harboring at least one of the 13 types targeted by the Digene HC2 "high-risk" HPV test and 33 cases containing "low-risk" HPV types (Table 1). The most prevalent genotype in the New Haven area was found to be HPV-16, followed by HPV-56. The combined rate of prevalence of HPV-16 and HPV-18 constituted 32.8% of the total isolates.

**Table 1:** HPV genotyping by DNA Sequencing v. Digene HC2 Test

*PCR/DNA Sequencing*			*Test Results by Digene HC2*	
	
Type Positive Cases		Prevalence (%)	High-risk+	Negative
16	29	(27.2)	11	18
56	9	(8.5)	9	0
31	7	(6.5)	5	2
6	6	(5.6)	2	4
18	6	(5.6)	6	0
54	6	(5.6)	3	3
58	6	(5.6)	5	1
66	6	(5.6)	6	0
59	4	(3.7)	4	0
45	3	(2.8)	2	1
83	3	(2.8)	2	1
32	2	(1.9)	0	2
35	2	(1.9)	1	1
39	2	(1.9)	2	0
40	2	(1.9)	0	2
52	2	(1.9)	1	1
33	1	(0.9)	1	0
53	1	(0.9)	1	0
62	1	(0.9)	1	0
68	1	(0.9)	1	0
70	1	(0.9)	1	0
72	1	(0.9)	0	1
73	1	(0.9)	0	1
M16	1	(0.9)	1	0
M 16, 18	1	(0.9)	1	0
M others	3	(2.9)	1	2
Total	107	(100)	67	40
			
			HC2 HPV detection rate = 67/107 = 62.6%	
Samples	513			
%HPV+ 20.9				

M16 = mixed infection with HPV 16 identified by type-specific sequencing primer.

M 16, 18 = mixed infection with HPV 16 and HPV 18 identified by type-specific sequencing primers.

M others = mixed infections by HPV types which cannot be sequenced with type-specific primers for HPV 6, 11, 16 or 18.

Digene High-risk + includes those cases reported as positive for both High-risk and Low-risk HPV types.

The underlined HPV genotypes are included in the "High-risk" Digene HC2 cocktail probe.

The HC2 high-risk HPV test identified only 11 (37.9%) of the 29 HPV-16 cases as positive. However, it successfully identified all the samples containing HPV-56 and HPV-18 as positive for high-risk HPV. The sensitivity of the HC2 test in detecting the predetermined "high-risk" HPV genotypes was summarized as follows:

HPV-18 100%

HPC-33 100%

HPV-39 100%

HPV-56 100%

HPV-59 100%

HPV-68 100%

HPV-58 83.3%

HPV-31 71.4%

HPV-45 66.7%

HPV-35 50%

HPV-52 50%

HPV-16 37.9%

Although HPV-54, -66, -83, -53 and -62 were not included in the hybridization cocktail probe, these genotypes were often reported to be positive for high-risk HPV by the HC2 test (Table 1).

Among the 107 nested PCR-positive samples, DNA sequencing with the GP6+ consensus general primer yielded multiple overlapping unreadable sequences in 5 cases. Using the individual type-specific primer sequencing for HPV-6,-11,-16 and -18 proved that one of them contained HPV-16, but not the other three genotypes, and that one contained a mixture of HPV-16 and HPV-18, but not the other two genotypes. For the remaining 3 mixed infection samples, repeated individual DNA sequencing failed to produce a readable primer extension/termination reaction with any of the four type-specific primers. Therefore, these latter 3 cases were considered to be multiple infections caused by HPVs other than the four vaccine-relevant types and grouped under the "low-risk" category.

Of the 513 cases studied, the Digene HC2 test classified 403 samples as negative and 75 as positive for "high-risk" HPV, and 35 as unsatisfactory for evaluation. These results were compared with those obtained by the nested PCR assay (Table 2). Since the Digene test only covered the "high-risk" HPV genotypes, specimens infected by HPV other than the 13 types targeted by the HC2 "high-risk" HPV cocktail probe would be classified as negative or unsatisfactory for evaluation (unsat.). In summary, HC2 test identified 50 of the 74 "high-risk" HPV-positive samples detected by nested PCR. The analytical sensitivity of the Digene HC2 test was calculated to be 50/74= 67.6% against the nested PCR assay. Since HC2 reported a total of 75 cases to be positive for "high-risk" HPV, its analytical specificity was calculated to be 50/75 = 66.7%. Two cases found to be unsatisfactory for PCR evaluation and negative by HC2 test were excluded from the total of 513 cases entered for analysis.

**Table 2:** Comparison of HPV nested PCR/sequencing and HC2 test results

		**PCR Results**				
		**Negative**	**High Risk**	**Low Risk**	**Unsat**.	**Total**

**HC2 Results**	**Negative**	364	23	16	0	403
	**High Risk**	8	50	17	0	75
	**Low Risk**	NP	NP	NP		
	**Unsat**.	34	1		0	35
	**Total**	406	74	33	0	513

**HC2**, Hybrid Capture 2; **HPV**, human papillomavirus; **PCR**, polymerase chain reaction.

**Unsat**. Test results or specimens considered inadequate for evaluation.

**High Risk **HPV types 16, 18, 31, 33, 35, 39, 45, 51, 52, 56, 58, 59 and 68.

**Low Risk **HPV types other than those listed as High Risk.

NP = not performed.

Data are given as number of specimens

## Discussion

The moderately thermostable LoTemp™ HiFi^® ^DNA polymerases are genetically engineered derivatives of a *Bacillus stearothermophilus (Bst) *DNA polymerase which was first introduced to resolve the hairpin structure in classic Sanger DNA sequencing by Ye and Hong [33]. *Bst *DNA polymerase with an optimum primer extension temperature at 65°C is extremely stable, capable of retaining its sequencing quality in working solution at room temperature for several weeks under conditions of robotic automation [34]. Modification of the amino acid sequence of a natural *Bst *DNA polymerase by site-directed genetic mutations increases the heat tolerance of the enzyme [35], which has paved the way to development of new thermostable DNA polymerases for repeated primer extension reactions under 85°C. The LoTemp™ HiFi^® ^DNA polymerase PCR protocol exploits the proof-reading and high-processivity properties of the modified *Bst *DNA polymerases at reduced cyclic temperatures.

Using LoTemp™ HiFi^® ^DNA polymerase for HPV DNA amplification eliminates all template purification steps which are usually required before PCR or sequencing reaction [21,22,32]. Since the ready-to-use polymerase mixture contains all the required ingredients for PCR, the need for in-house pipetting is minimal. Since the DNA polymerase and other reagent components are stabilized for storage at room temperature, there is no need to keep ice-cold blocks while setting up the PCR. Since this system uses chemical melting agents for dsDNA denaturing and a high-processivity DNA polymerase for nucleotide primer extension under partial isostabilization, it allows thermocycling at 85°C for denaturing, 40°C for annealing and 65°C for primer extension, respectively. At lowered cycling temperatures, the rate of heat-induced mutations [36], namely depurination [37] and deamination [38] of the nitrogenous bases, in the DNA molecules during PCR amplification is reduced. As a result, the PCR products are more homogeneous.

In this report, we have demonstrated that the LoTemp™ PCR system can amplify 1–10 copies of purified HPV-16, -18 or -6B to generate a corresponding type-specific 150 bp nested PCR product. The sensitivity of LoTemp™ PCR in the detection of plasmid HPV DNA is about 10× greater than that obtained by the traditional heat-resistant *Taq *DNA polymerases without nested amplification (Fig. 1). Nested PCR has increased the analytical sensitivity of HPV DNA detection on cervicovaginal cell suspensions [21,22,32], which is also our experience (Fig. 2). Nested PCR also serves to eliminate most of the interfering substances which otherwise may have to be removed by column purification in the procedure.

Although the MY09/MY11 and GP5+/GP6+ consensus general primer pairs have been used to amplify the highly conserved L1 gene region of all HPV genotypes, their efficiency in target DNA amplification varies from one HPV genotype to another with discordant patterns [22]. We have found that some isolates of HPV-39 and HPV-73 are not amplifiable by the GP5+/GP6+ primer pair either in primary PCR or in nested PCR. A second amplification with a GP5+/MY09 and a GP6+/MY11 primer pair generates a longer and shorter PCR product, respectively. The GP6+/MY11 heminested PCR product (Fig. 3) is suitable for direct DNA sequencing. GP5+/GP6+ failure in amplification of HPV-39 [39] and other HPV types [22,32] in clinical specimens is a well-known technical problem if this single pair of primers is relied upon for HPV detection. However, GP5+ and GP6+ are excellent nested PCR primers for preparing templates for HPV genotyping with direct DNA sequencing.

Optimization of the PCR protocol is essential for detection of HPV. Without optimization, a PCR method using one set of consensus primers for amplification may have a lower sensitivity than the Digene HC2 test [40]. Using an initial 40-cycle amplification on clinical specimens, Johnson *et al*. [21] reported that nested PCR generally increases the sensitivity of an HPV PCR method by about 60%. With the PCR protocol presented in this paper, this difference is augmented due to adoption of a 30-cycle amplification. An advantage of reducing the number of amplification cycles is that co-amplification of non-specific interfering DNA molecules is reduced in the primary PCR in favor of generating specific nested PCR products for direct DNA sequencing.

Using our optimized protocol, nested PCR detected 107 HPV-positive cases among 513 clinical samples (Table 1). Twenty-three (23) HPV genotypes have been identified, including 12 of the 13 "high-risk" genotypes, i.e. HPV-16, -18, -31, -33, -35, -39, -45, -52, -56, -58, -59, and -68, which are targeted by the Digene HC2 test. The lack of representation of HPV-51 which is also targeted by the HC 2 test probably reflects a low regional prevalence of this genotype. Using the same primers for nested PCR followed by genotyping with DNA sequencing, HPV-51 was found to be a relatively common genotype in Germany, constituting about 5% of the total HPV isolates detected [22]. However, it was not recorded even once among 894 HPV isolates in Denmark [21].

The hypervariable region of the DNA sequence downstream of the GP5+ binding site is critical in L1 genotyping of HPV. While most HPV genotypes can be determined through alignment of a 34-bp sequence in this region with the GenBank database [32], accurate genotyping may require alignment of a longer sequence, for example 50 bp long, when the result of BLAST algorithm is ambiguous. If a 34-bp sequence is relied upon for genotyping, the number of HPV-16 infections may be under-estimated.

For the development of this protocol, we found that using 1 μL of 5 μmolar OPC-purified GP6+ oligonucleotide instead of a manufacturer-recommended 3.2 μmolar solution as the sequencing primer seems to generate more consistent sequencing results for typing various HPV isolates. The concentration of primer may need to be adjusted if a higher grade of GP6+ is used as the DNA sequencing primer.

Of the 107 PCR-positive samples, the Digene HC2 test has classified 67 as HPV-positive, a detection rate of 62.6% (Table 1). When only the HC2-targeted "high-risk" HPV genotypes are used for comparison, nested PCR detects 74 HPV-positive cases while HC2 test identifies 50 in this group with an analytical sensitivity of 67.6% (50/74).

The most prevalent is HPV-16, constituting 27.2% of the total isolates (Table 1). This percentage of HPV-16 prevalence is almost identical to those reported by others using MY/GP nested PCR/DNA sequencing genotyping on cervicovaginal cell suspensions, e.g. 26% in Denmark [21] and 26.2% in Germany [22]. Digene HC2 test identified 11 of the 29 HPV-16 PCR-positive cases with a detection rate of 37.9% which is surprisingly low. Since all the HPV-16 PCR-positive results have been validated by DNA sequencing for genotyping with a prevalence rate similar to those commonly found in the Western world based on the same methodology [21,22] and since the Digene HC2 tests were performed by two independent clinical laboratories properly certified by the health authorities, the validity of the individual test results seems not to be in question. The discrepancy is probably due to the fact that there are numerous HPV-16 sequence variants in a given patient population [41], which may not be all targeted by the HC2 RNA cocktail probe, but share a highly conserved region of the L1 gene that the MY09/MY11 primers amplify effectively. In support of this interpretation is the fact that the HC2 test has correctly identified all cases of HPV-18, HPV-39, HPV-56 and HPV-59 as "high-risk" in the same data.

HPV-56 is the second most prevalent high-risk genotype detected (8.5%), followed by HPV-31, -18, -54, -58 and -66, sharing about the same rate of prevalence (5.6–6.5%). The combined number of HPV-16 and -18 cases constitutes only 32.8% of the single HPV isolates in our series. HPV-18 also seems to play a relatively minor role in causing cervical pathology in Canada [42].

It has been reported by others [43] that the Digene HC2 high-risk test may be able to detect HPV types -53, -54, -62, -66 and -83 and label them as high-risk HPVs although these genotypes are not intentionally targeted in its high-risk cocktail probe. Our findings confirm these cross-reactions (Table 1). Sequence variation within the probe binding sites [44] and non-specific binding between the probe and non-targeted mismatched DNA [45-48] are well recognized sources of error if nucleic acid hybridization is relied upon for microbial and viral genotyping. When the GP5+/GP6+ PCR products with a hypervariable DNA sequence are targeted for developing a multiplex genotyping method [49], the DNA probe designed for HPV-66, a recently recognized high-risk type [50-52], is found to react with HPV-52 and the probe for HPV-82 with HPV-51 due to cross-hybridization despite the presence of four base mismatches in each pair. Some experts [40] consider the unintended cross-reactions of the Digene HC2 test with non-targeted HPV types, such as HPV-66, which occasionally may cause cancer, to be "fortunate". However, the benefit to the patients of these cross-reactions needs additional confirmation.

Fifty percent (50%) of the HPV-54 isolates are returned by HC2 test as high-risk HPV. HPV-54 has been classified as a low-risk virus [51,53], but is found to be associated with a 40-fold increase in risk among American Indian women with CIN 2/3. Only HPV-16 has shown a higher risk than HPV-54 among this subpopulation [54]. Genetic make-up of a patient may have to be considered in using HPV genotyping information for the follow-up of persistent infections [55].

For the mixed infection cases, we choose single primers specific for HPV-6, -11, -16 and -18 [31] to perform individual specific primer DNA sequencing in order to determine if the mixed infection includes any of these vaccine-relevant HPV types. The rationale for this choice is that the majority of multiple HPV infections are transient [6-15]. The immediate concern to the patient and her health care provider is whether the mixed infection is caused by any of the vaccine-relevant HPV types if the patient is considering vaccine immunization.

The low percentage (< 5%) of multiple HPV infections observed in our series might have been biased because a large proportion of the specimens for this study was collected from a solo private practitioner's office and this group of specimens had an exceptionally low positive rate and no multiple HPV infections at all. The rate of multiple HPV infections is known to vary among patient populations and is also influenced by the stage of carcinogenesis. Multiple HPV infections were found in less than 5% of the HPV-positive samples from patients with invasive cancer lesions, but over 15% of the positive samples in the control group [27]. Multiple HPV infections tend to evolve into single HPV infections as the infection becomes chronic and persistent while the cervical cytopathology progresses from metaplasia, LSIL, HSIL, carcinoma-in-situ to invasive cancer [56].

In summary, we have reported our experience in adapting the well characterized PCR/DNA sequencing protocol for routine HPV genotyping. We believe that a sensitive and specific HPV assay followed by genotyping with direct DNA sequencing will generate useful information for following persistent infections while referring the patients at "high-risk" of developing HSIL, not the patients with a "high-risk HPV", to colposcopic evaluation.

## Conclusion

The nested PCR technology using MY09/MY11 and GP5+/GP6+ consensus primers for target HPV DNA amplification can be used in diagnostic laboratories for routine HPV detection and to prepare clinical materials for genotyping by direct DNA sequencing. We have adapted a newly introduced low-temperature PCR system and optimized the protocol to facilitate the transfer of this molecular technology into clinical laboratory practice for accurate HPV genotyping which is a valuable tool for follow-up of patients with persistent HPV infection, a recognized tumor promoter in cancer induction.

## References

[B1] Kjaer SK, van den Brule AJ, Bock JE, Poll PA, Engholm G, Sherman ME, Walboomers JM, Meijer CJ (1996). Human papillomavirus- the most significant risk determinant of cervical intraepithelial neoplasia. Int J Cancer.

[B2] (2005). HC2 HPV DNA Test-REF 5199-1220 Digene Corporation, Gaithersburg, MD 20878 USA.

[B3] Gogola J, Van Dinh T, Lucci JA, Smith E, Hannigan EV (2001). Human papillomavirus testing for triage in a referral population. J Low Genit Tract Dis.

[B4] ACOG Practice Bulletin No 61 (2005). Human Papillomavirus. Obstet Gynecol.

[B5] Wu HH, Allen SL, Kirkpatrick JL, Elsheikh TM (2006). Reflex high-risk human papilloma virus DNA test is useful in the triage of women with atypical squamous cells cannot exclude high-grade squamous intraepithelial lesion. Diagn Cytopathol.

[B6] Wallin KL, Wiklund F, Angstrom T, Bergman F, Stendahl U, Wadell G, Hallmans G, Dillner J (1999). Type-specific persistence of human papillomavirus DNA before the development of invasive cervical cancer. N Engl J Med.

[B7] Kjaer SK, van den Brule AJ, Paull G, Svare EI, Sherman ME, Thomsen BL, Suntum M, Bock JE, Poll PA, Meijers CJ (2002). Type specific persistence of high risk human papillomavirus (HPV) as indicator of high grade cervical squamous intraepithelial lesions in young women: population based prospective follow up study. BMJ.

[B8] Cuschieri KS, Cubie HA, Whitley MW, Gilkison G, Arends MJ, Graham C, McGoogan E (2005). Persistent high risk HPV infection associated with development of cervical neoplasia in a prospective population study. J Clin Pathol.

[B9] Brummer O, Hollwitz B, Bohmer G, Kuhnle H, Petry KU (2006). Human papillomavirus-type persistence patterns predict the clinical outcome of cervical intraepithelial neoplasia. Gynecol Oncol.

[B10] Jacobs MV, Walboomers JM, Snijders PJ, Voorhorst FJ, Verheijen RH, Fransen-Daalmeijer N, Meijer CJ (2000). Distribution of 37 mucosotropic HPV types in women with cytologically normal cervical smears: the age-related patterns for high-risk and low-risk types. Int J Cancer.

[B11] Herrero R, Hildesheim A, Bratti C, Sherman ME, Hutchinson M, Morales J, Balmaceda I, Greenberg MD, Alfaro M, Burk RD, Wacholder S, Plummer M, Schiffman M (2000). Population-based study of human papillomavirus infection and cervical neoplasia in rural Costa Rica. J Natl Cancer Inst.

[B12] Molano M, Posso H, Weiderpass E, van den Brule AJ, Ronderos M, Franceschi S, Meijer CJ, Arslan A, Munoz N, HPV Study Group HPV Study (2002). Prevalence and determinants of HPV infection among Colombian women with normal cytology. Br J Cancer.

[B13] Rousseau MC, Pereira JS, Prado JC, Villa LL, Rohan TE, Franco EL (2001). Cervical coinfection with human papillomavirus (HPV) types as a predictor of acquisition and persistence of HPV infection. J Infect Dis.

[B14] Hinchliffe SA, van Velzen D, Korporaal H, Kok PL, Boon ME (1995). Transience of cervical HPV infection in sexually active, young women with normal cervicovaginal cytology. Br J Cancer.

[B15] Brown DR, Shew ML, Qadadri B, Neptune N, Vargas M, Tu W, Juliar BE, Breen TE, Fortenberry JD (2005). A longitudinal study of genital human papillomavirus infection in a cohort of closely followed adolescent women. J Infect Dis.

[B16] Cuschieri KS, Cubie HA, Whitley MW, Seagar AL, Arends MJ, Moore C, Gilkisson G, McGoogan E (2004). Multiple high risk HPV infections are common in cervical neoplasia and young women in a cervical screening population. J Clin Pathol.

[B17] Wensveen CW, Kagie MJ, Nagelkerke NJ, Veldhuizen RW, Trimbos JB (2005). Can viral load, semi-quantitatively evaluated, of human papillomavirus predict cytological or histological outcome in women with atypical squamous or glandular cells of undetermined significance cytology?. Eur J Gynaecol Oncol.

[B18] Sherman ME, Wang SS, Wheeler CM, Rich L, Gravitt PE, Tarone R, Schiffman M (2003). Determinants of human papillomavirus load among women with histological cervical intraepithelial neoplasia 3 : dominant impact of surrounding low-grade lesions. Cancer Epidemiol Biomarkers Prev.

[B19] Castle PE, Schiffman M, Burk RD, Wacholder S, Hildesheim A, Herrero R, Bratti MC, Sherman ME, Lorincz A (2002). Restricted Cross-Reactivity of Hybrid Capture 2 with Nononcogenic Human Papillomavirus Types. Cancer Epidemiol Biomarkers & Prev.

[B20] Vernon SD, Unger ER, Williams D (2000). Comparison of human papillomavirus detection and typing by cycle sequencing, line blotting, and hybrid capture. J Clin Microbiol.

[B21] Johnson T, Bryder K, Corbet S, Fomsgaard A (2003). Routine genotyping of human papillomavirus samples in Denmark. APMIS.

[B22] Speich N, Schmitt C, Bollmann R, Bollmann M (2004). Human papillomavirus (HPV) study of 2916 cytological samples by PCR and DNA sequencing : genotype spectrum of patients from the west German area. J Med Microbiol.

[B23] Kosel S, Burggraf S, Mommsen J, Engelhardt W, Olgemoller B (2003). Type-specific detection of human papillomaviruses in a routine laboratory setting–improved sensitivity and specificity of PCR and sequence analysis compared to direct hybridisation. Clin Chem Lab Med.

[B24] Lonky NM, Felix JC, Naidu YM, Wolde-Tsadik G (2003). Triage of atypical squamous cells of undetermined significance with hybrid capture II: colposcopy and histologic human papillomavirus correlation. Obstet Gynecol.

[B25] Stoler MH, Castle PE, Solomon D, Schiffman M (2007). The Expanded Use of HPV Testing in Gynecologic Practice per ASCCP-Guided Management Requires the Use of Well-Validated Assays. Am J Clin Pathol.

[B26] VRBPAC Background Document Gardasil™ HPV Quadrivalent Vaccine. VRBPAC Meeting.

[B27] Asato T, Maehama T, Nagai Y, Kanazawa K, Uezato H, Kariya K (2004). A large case-control study of cervical cancer risk associated with human papillomavirus infection in Japan, by nucleotide sequencing-based genotyping. J Infect Dis.

[B28] http://www.uri.edu/research/gsc/resources/cndna.html.

[B29] Protocols used at NCI & Related Information-Processing of Microdissected Tissue for Molecular Analysis. http://cgap-mf.nih.gov/Protocols/Processing/DNA.html.

[B30] Ayatollahi M, Zakerinia M, Haghshenas M (2005). Molecular analysis of Iranian families with sickle cell disease. J Trop Pediatr.

[B31] Gharizadeh B, Oggionni M, Zheng B, Akom E, Pourmand N, Ahmadian A, Wallin KL, Nyren P (2005). Type-specific multiple sequencing primers: a novel strategy for reliable and rapid genotyping of human papillomaviruses by pyrosequencing technology. J Mol Diagn.

[B32] Feoli-Fonseca JC, Oligny LL, Filion M, Brochu P, Simard P, Russo PA, Yotov WV (1998). A two-tier polymerase chain reaction direct sequencing method for detecting and typing human papillomaviruses in pathological specimens. Diagn Mol Pathol.

[B33] Ye S, Hong G (1987). Heat-stable DNA polymerase I large fragment resolves hairpin structure in DNA sequencing. Scientia sinica (Series B).

[B34] Earley JJ, Kuivaniemi H, Prockop DJ, Tromp G (1994). Robotic automation of dideoxyribonucleotide sequencing reactions. Biotechniques.

[B35] Hong GF, Huang W DNA polymerase having ability to reduce innate selective discrimination against fluorescent dye-labeled dideoxynucleotides. US Patent.

[B36] Andre P, Kim A, Khrapko K, Thilly WG (1997). Fidelity and mutational spectrum of Pfu DNA polymerase on a human mitochondrial DNA sequence. Genome Res.

[B37] Lindahl T, Nyberg B (1972). Rate of depurination of native deoxyribonucleic acid. Biochemistry.

[B38] Lindahl T, Nyberg B (1974). Heat-induced deamination of cytosine residues in deoxyribonucleic acid. Biochemistry.

[B39] Evans MF, Adamson CS, Simmons-Arnold L, Cooper K (2005). Touchdown General Primer (GP5+/GP6+) PCR and optimized sample DNA concentration support the sensitive detection of human papillomavirus. BMC Clin Pathol.

[B40] Schiffman M, Wheeler CM, Dasgupta A, Solomon D, Castle PE (2005). The ALTS Group. A comparison of a prototype PCR assay and hybrid capture 2 for detection of carcinogenic human papillomavirus DNA in women with equivocal or mildly abnormal Papanicolaou smears. Am J Clin Pathol.

[B41] Tornesello ML, Duraturo ML, Salatiello I, Buonaguro L, Losito S, Botti G, Stellato G, Greggi S, Piccoli R, Pilotti S, Stefanon B, De Palo G, Franceschi S, Buonaguro FM (2004). Analysis of human papillomavirus type-16 variants in Italian women with cervical intraepithelial neoplasia and cervical cancer. J Med Virol.

[B42] Feoli-Fonseca JC, Oligny LL, Brochu P, Simard P, Falconi S, Yotov WV (2001). Human papillomavirus (HPV) study of 691 pathological specimens from Quebec by PCR-direct sequencing approach. J Med Virol.

[B43] Ortiz M, Torres M, Munoz L, Fernandez-Garcia E, Canals J, Cabornero AI, Aguilar E, Ballesteros J, Del Amo J, Garcia-Saiz A (2006). Oncogenic human papillomavirus (HPV) type distribution and HPV type 16 E6 variants in two Spanish population groups with different levels of HPV infection risk. J Clin Microbiol.

[B44] Anderson TP, Werno AM, Beynon KA, Murdoch DR (2003). Failure To Genotype Herpes Simplex Virus by Real-Time PCR Assay and Melting Curve Analysis Due to Sequence Variation within Probe Binding Sites. J Clin Microbiol.

[B45] Goris J, Konstantinidis KT, Klappenbach JA, Coenye T, Vandamme P, Tiedje JM (2007). DNA-DNA hybridization values and their relationship to whole-genome sequence similarities. Int J Syst Evol Microbiol.

[B46] Huang CC, Qiu JT, Kashima ML, Kurman RJ, Wu TC (1998). Generation of type-specific probes for the detection of single-copy human papillomavirus by a novel in situ hybridization method. Mod Pathol.

[B47] Naef F, Lim DA, Patil N, Magnasco M (2002). DNA hybridization to mismatched templates: a chip study. Phys Rev E Stat Nonlin Soft Matter Phys.

[B48] Sorokin NV, Chechetkin VR, Livshits MA, Pan'kov SV, Donnikov MY, Gryadunov DA, Lapa SA, Zasedatelev AS (2005). Discrimination between perfect and mismatched duplexes with oligonucleotide gel microchips: role of thermodynamic and kinetic effects during hybridization. J Biomol Struct Dyn.

[B49] Schmitt M, Bravo IG, Snijders PJ, Gissmann L, Pawlita M, Waterboer T (2006). Bead-based multiplex genotyping of human papillomaviruses. J Clin Microbiol.

[B50] Tawheed AR, Beaudenon S, Favre M, Orth G (1991). Characterization of human papillomavirus type 66 from an invasive carcinoma of the uterine cervix. J Clin Microbiol.

[B51] Munoz N, Bosch FX, de Sanjose S, Herrero R, Castellsague X, Shah KV, Snijders PJ, Meijer CJ (2003). International Agency for Research on Cancer Multicenter Cervical Cancer Study Group: Epidemiologic classification of human papillomavirus types associated with cervical cancer. N Engl J Med.

[B52] Prado JC, Calleja-Macias IE, Bernard HU, Kalantari M, Macay SA, Allan B, Williamson AL, Chung LP, Collins RJ, Zuna RE, Dunn ST, Ortiz-Lopez R, Barrera-Saldana HA, Cubie HA, Cuschieri K, von Knebel-Doeberitz M, Sanchez GI, Bosch FX, Villa LL (2005). Worldwide genomic diversity of the human papillomaviruses-53, 56, and 66, a group of high-risk HPVs unrelated to HPV-16 and HPV-18. Virology.

[B53] Favre M, Kremsdorf D, Jablonska S, Obalek S, Pehau-Arnaudet G, Croissant O, Orth G (1990). Two new human papillomavirus types (HPV54 and 55) characterized from genital tumours illustrate the plurality of genital HPVs. Int J Cancer.

[B54] Schiff M, Becker TM, Masuk M, van Asselt-King L, Wheeler CM, Altobelli KK, North CQ, Nahmias AJ (2000). Risk factors for cervical intraepithelial neoplasia in southwestern American Indian women. Am J Epidemiol.

[B55] Trimble CL, Piantadosi S, Gravitt P, Ronnett B, Pizer E, Elko A, Wilgus B, Yutzy W, Daniel R, Shah K, Peng S, Hung C, Roden R, Wu TC, Pardoll D (2005). Spontaneous regression of high-grade cervical dysplasia: effects of human papillomavirus type and HLA phenotype. Clin Cancer Res.

[B56] Zuna RE, Allen RA, Moore WE, Mattu R, Dunn ST (2004). Comparison of human papillomavirus genotypes in high-grade squamous intraepithelial lesions and invasive cervical carcinoma : evidence for differences in biologic potential of precursor lesions. Modern Pathology.

